# The Housing Experiment: Citizen Science Engaging Older Adults and School Pupils to Assess Housing Accessibility

**DOI:** 10.1093/geroni/igaa057.2991

**Published:** 2020-12-16

**Authors:** Susanne Iwarsson, Fredrik Brounéus, Knut mårtensson, Marianne Granbom

**Affiliations:** 1 Lund University, Lund, Skane Lan, Sweden; 2 Vetenskap & Allmänhet, Stockholm, Stockholms Lan, Sweden; 3 miThings, Lund, Skane Lan, Sweden; 4 Lund University, Lund, Sweden

## Abstract

Citizen science is gaining momentum as an approach in many scientific fields. However, it is scarcely used in aging research. Since 2009 in Sweden, Public & Science (NGO) has coordinated an annual mass-experiment where thousands of school pupils have collected data that would have been impossible for researchers to collect on their own. Designed as a cross-generational endeavour, the 2020 mass-experiment, the Housing Experiment, is based on scientific methodology for data collection on housing accessibility. The aim of this presentation is to describe the iterative development process involving older adults, stakeholders in the housing sector, teachers and pupils. We will present the app and instructions developed and piloted for data collection, as well as usability and interrater reliability results. With media attention already in the planning phases, this citizen science initiative has the potential to both generate a unique dataset and increase engagement in housing matters concerning the aging population.

